# High speciation rate of niche specialists in hot springs

**DOI:** 10.1038/s41396-023-01447-4

**Published:** 2023-06-07

**Authors:** Qing He, Shang Wang, Kai Feng, Sean T. Michaletz, Weiguo Hou, Wenhui Zhang, Fangru Li, Yidi Zhang, Danrui Wang, Xi Peng, Xingsheng Yang, Ye Deng

**Affiliations:** 1 CAS Key Laboratory for Environmental Biotechnology, Research Center for Eco-Environmental Sciences, Chinese Academy of Sciences (CAS), Beijing, 100085 China; 2 College of Resources and Environment, University of Chinese Academy of Sciences, Beijing, 100190 China; 3 Department of Botany and Biodiversity Research Centre, University of British Columbia, Vancouver, BC V6T 1Z4 Canada; 4 State Key Laboratory of Biogeology and Environmental Geology, China University of Geosciences, Beijing, 100083 China

**Keywords:** Microbial ecology, Microbial ecology, Phylogenetics, Microbial ecology

## Abstract

Ecological and evolutionary processes simultaneously regulate microbial diversity, but the evolutionary processes and their driving forces remain largely unexplored. Here we investigated the ecological and evolutionary characteristics of microbiota in hot springs spanning a broad temperature range (54.8–80 °C) by sequencing the 16S rRNA genes. Our results demonstrated that niche specialists and niche generalists are embedded in a complex interaction of ecological and evolutionary dynamics. On the thermal tolerance niche axis, thermal (T) sensitive (at a specific temperature) versus T-resistant (at least in five temperatures) species were characterized by different niche breadth, community abundance and dispersal potential, consequently differing in potential evolutionary trajectory. The niche-specialized T-sensitive species experienced strong temperature barriers, leading to completely species shift and high fitness but low abundant communities at each temperature (“home niche”), and such trade-offs thus reinforced peak performance, as evidenced by high speciation across temperatures and increasing diversification potential with temperature. In contrast, T-resistant species are advantageous of niche expansion but with poor local performance, as shown by wide niche breadth with high extinction, indicating these niche generalists are “jack-of-all-trades, master-of-none”. Despite of such differences, the T-sensitive and T-resistant species are evolutionarily interacted. Specifically, the continuous transition from T-sensitive to T-resistant species insured the exclusion probability of T-resistant species at a relatively constant level across temperatures. The co-evolution and co-adaptation of T-sensitive and T-resistant species were in line with the red queen theory. Collectively, our findings demonstrate that high speciation of niche specialists could alleviate the environmental-filtering-induced negative effect on diversity.

## Introduction

Temperature is a key driver of microbial diversity in geothermal ecosystems [1–3]. Although it is well established that microbial diversity correlated with temperature [4–6], efforts to understand the mechanisms by which temperature regulates diversity have yielded two perspectives. In the first, diversity is the outcome of ecological processes largely through the effects of temperature on compositional turnover of extant species, due to interspecific differences [7] in thermal tolerance (the temperature range within which a species can grow), but may also be a consequence of species interactions [8]. In the second, diversity originates from evolutionary processes, primarily as a consequence of temperature effects on speciation and/or extinction rates [9, 10]. These ecological and evolutionary processes commonly co-occur and contribute simultaneously [11, 12] in the same context, but the evolutionary processes are largely unexplored relative to the ecological processes [13, 14].

Speciation is the ultimate driver of biodiversity, and understanding the factors influencing rates of speciation and its feedback on species richness is a central challenge in ecology. Specific prediction about how ambient temperature should relate to species richness was developed in the context of metabolic theory of ecology (MTE) for macrobes [15, 16] and later was extended to microbes [17, 18]. These previous studies mainly focus on temperature gradients not exceeding 45 °C. Recent analyses documented that metabolic theory of ecology holds true for mesophiles (temperature optima ≤ 45 °C), but not for thermophiles (>45 °C) when considering temperature-dependence of growth rate and suggested activation energy of *E* = 0.65 eV for mesophiles and *E* ≈ 0 eV for thermophiles [19]. One reason that thermophilic metabolism may depart from the canonical MTE hypothesis is that they assumed constant biomass with temperature, but microbial biomass in hot springs also decreases exponentially with temperature [20]. For thermophiles, environmental temperature has been a major determinant of evolutionary rates [21, 22] and some studies have reported that thermophillic generation time was inversely related to temperature [23], which predicts higher speciation rates at higher temperatures. Simultaneously, higher temperatures would exclude microbial species with poor adaptation via harsh environmental filtering [5, 6], eventually showing a higher extinction rate. However, direct evidence for a temperature dependence of speciation and extinction rates from the phylogenetic perspective has not yet been shown.

Geothermal springs act as isolated islands of microbial evolution, and microbiota therein experience greater dispersal limitation [24] and faster evolution rates [25]. But even for these thermophiles, different species could have opposite responses to local conditions such as temperature. Niche breadth might act as an important evolutionary driver, influencing the rate of species diversification and adaptation [26, 27]. Species adapted to a wide or narrow temperature have different survivability [28], due to their differences in biochemical and physiological properties and ecological and evolutionary adaptability [29–32]. However, whether and how differences in niche breadth (i.e. thermal tolerance) have an impact on the ecological and evolutionary processes and consequently species diversity is largely unknown.

In this study, the ecological and evolutionary processes of microbiota over a wide temperature range of 54.8–80 °C were characterized based on high-throughput sequencing of 16S rRNA genes. BiSSE (binary-state speciation and extinction) model was employed to characterize evolutionary features in microbial adaptation to high temperature. We asked the following questions: (i) How niche breadth (especially on the temperature niche axis) influence the ecological and evolutionary performance? (ii) How niche specialists and niche generalists interacted evolutionarily across temperatures? (iii) How ecological and evolutionary trade-offs on community-level influence the overall diversity pattern in the face of increasing environmental extremes (i.e., temperature)?

## Methods and materials

### Field measurements and sample collection

Field measurements and sample collections were carried out in August 2019 in Tengchong, Yunnan Province, China (N 24° 56′~25° 27′, E 98° 26′~98° 27′) (Fig. S1). The study site was situated at the previously described Rehai geothermal fields [2, 6], full of intense hydrothermal activity with numerous springs and mud pools. Direchi (DRC) was chosen to collect samples along the flowing path with temperature decreasing from 80 °C at the vent down to 54.8 °C in a pool. The overlaying water on the sediment is just 5–15 cm deep. The in-situ temperature and pH were measured by immersing a portable temperature sensor (Hl9124, Hanna Instruments, Italy) in the surface water just above the sediments. This flow path could be divided into different small-scale eco-regions by the colorful floating microbial mat and we collected surface sediment samples for microbial and chemical analyses from eight small-scale eco-regions labeled DRC1 (80 °C) to DRC8 (54.8 °C), each has seven randomly distributed replicates (Fig. S1). These samples were collected with sterile spatulas and spoons, and homogenized in a pre-sterilized aluminum pan before being placed into tubes. Additionally, the concentrations of nitrite (NO_2_^−^), sulfate (SO_4_^2−^), hydrogen sulfide (H_2_S), ferrous iron (Fe^2+^), total iron (Fe_total_), and dissolved oxygen (DO) at each sampling region were measured in-situ using Hach test kits (Hach Chemical Co., IA, USA) (Table S1). Total Nitrogen (TN), nitrate (NO_3_^−^) and organic matter (OM) were measured on air-dried sediments, according to a previously published protocol [33] (Table S1). All samples were immediately frozen on dry ice and stored at −80 °C in the laboratory until further analysis.

### DNA extraction, amplification of FL and V4 region of 16S rRNA gene and sequencing

Nucleic acids were extracted from 0.25 g of sediment using the MoBio Power Soil DNA isolation kit (MoBio Laboratories, Carlsbad, CA, USA) according to the manufacture’s protocol. Each sample was done in triplicates and extracted DNAs for each sample were pooled into one collection tube at final step. The full-length (FL) and V4 region 16S rRNA genes sequences were amplified using the primer set 27F (5′-AGRGTTYGATYMTGGCTCAG-3′)/1492R (5′-RGYTACCTTGTTACGACTT-3′) and 515F (5′-GTGYCAGCMGCCGCGGTAA-3′)/806R (5′-GGACTACHVGGGTWTCTAAT-3′) with unique barcode sequences at both 5′ ends, respectively. The FL PCR system was performed in 30 μl mixture containing 10.5 μl NFW, 15 μl KOD ONE MM, 1.5 μl forward and reverse primers (10 μM) and 1.5 μl of template DNA (5~30 ng). Each 50 μl PCR amplification mixture of V4 region contained 5 μl 10× PCR buffer, 1.5 μl dNTP mixture (10 mM for each), 1.5 μl forward and reverse primers (10 μM), 0.5 μl Taq DNA Enzyme (TaKaRa), 2 μl DNA, 1 μl BSA, and 37 μl ddH2O. The PCR program was as follows: 95 °C for 5 min (3 min for V4 region), 30 cycles of 95 °C for 30S (15S for V4), 55 °C for 30S (15S for V4) and 72 °C for 90S (45S for V4), and final extension at 72 °C for 7 min (5 min for V4). The PCR products were verified by 1.2% (1.8% for V4) agarose gels and purified with the Monarch DNA Gel Extraction Kit. The concentrations were quantified with Qubit fluorimeter (Invitrogen, Carlsbad, CA), and then equal molar amounts of DNA were pooled for PacBio and HiSeq library construction and then sent for sequencing on the PacBio RS II platform [34] at Biomarker Biotechnology Co., Ltd. (Beijing, China) and HiSeq platform at Magigene Biotechnology Co., Ltd (Guangzhou, China), respectively.

### Absolute quantification of bacterial biomass by ddPCR

The copy numbers of 16S rRNA for bacteria were quantified by droplet digital PCR (ddPCR) with a probe approach [35]. Triplicates of 20 μl ddPCR amplification mixtures, composed of 10 μl ddPCR supermix for probes, 1.8 μl forward primer 515 F (10 μM) (5′-GTGYCAGCMGCCGCGGTAA-3′), 1.8 μl reverse primer 926 R (10 μM) (5′-CCGYCAATTYMTTTRAGTTT-3′), 0.5 μl bacterial-probe (10 μM) (5′FAM-ACTACNVGGGTWTCTAATCCBKTT-BHQ3′), 2 μl of template DNA (5~30 ng), and 3.9 μl ddH_2_O, were converted to 12,000–20,000 droplets using the QX200 droplet generator (Bio-Rad). The generated droplets for each sample were then transferred to a 96-well plate and amplified in a MyCycler thermocycler (Bio-Rad) using the following conditions: 10 min at 95 °C; 40 cycles of denaturing at 94 °C for 30S, annealing at 47 °C for 30S, extension at 72 °C for 1 min; and a final extension at 98 °C for 10 min. Subsequently, the plate was loaded onto the QX200 droplet digital reader (Bio-Rad), which automatically reads the droplets from each well of the plate. Data were analyzed using QuantaSoft software (Bio-Rad) and corrected for the various amounts of template DNA used.

### Sequence processing and statistical analysis

**Fig. 1:**
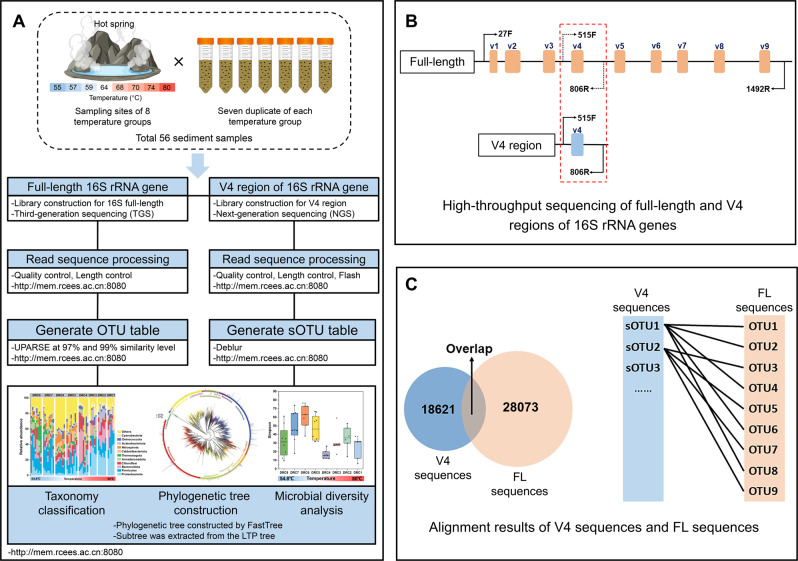
The experimental design and analytical workflow. **A** Data processing for the full-length sequencing and V4 region sequencing. **B** Primer set used for targeting full-length 16S rRNA genes and its hypervariable V4 regions. **C** The overlap OTUs between V4 sequences and full-length sequences and mapping V4 sequences’ sOTUs to full-length sequences’ OTUs.

The short reads (V4 region) generated by high-throughput sequencing were analyzed via an in-house Galaxy Pipeline (http://mem.rcees.ac.cn:8080) [36] (Fig. 1A). Briefly, the raw sequences were demultiplexed by barcode identification with no errors allowed. Then primer sequences were trimmed and forward and reverse reads were joined using FLASH [37], followed by quality control. Quality filtering criteria included average quality score >20, minimum length of 140 bp, and no ambiguous bases. Good quality reads were subjected to generate sub-operational-taxonomic-unit (sOTU, equal to amplicon sequence variant (ASV)) using Deblur [38] (Fig. 1A). The raw PacBio FL sequences were initially subjected to correct sequence errors using the JGI SMRT Portal “reads of insert” protocol with accuracy >99%, corresponding to Q20. Then, quality filtering, chimera detection and clustering were also performed via the Galaxy Pipeline mentioned above (Fig. 1A). Reads ≤1340 bp or ≥1640 bp were removed based on read length analysis [39].

Currently, the sequences processing methods for FL reads of 16S rRNA gene are not as mature as the analysis of short reads. Different methods are used for clustering long-reads, some based on ASVs [40, 41], others based on OTUs either at 99% [42] or 97% similarity level [43]. Undoubtedly, exact ASVs are providing single-nucleotide resolution and are independent of a 16S rRNA gene reference database [44]. However, the use and development of ASV methods are mostly based on short reads, careful consideration could be taken when applied to long-reads. The big concern is the considerable error rate of long-reads sequencing based on PacBio CCS [45] and cluster-free method of ASVs will undoubtedly amplify the impact of these sequencing errors. In our study, we tried to control the artificial inflation of diversity estimates, and thus it is necessary to bin similar sequences together (i.e. UPARSE) to mitigate the impact of sequencing errors [39, 42, 43]. By using UPARSE [46] (Fig. 1A), we tested two clustering thresholds, at 99% and 97% similarity level. The results suggested that the clustering thresholds at 99% and 97% resulted in similar trends for microbial taxonomic diversity and phylogenetic diversity (Pearson correlation, *R*^2^ = 0.36~0.79, *p* < 0.001), but 99% similarity level had far exceeding number of rare species. We finally chose at 97% similarity level to cluster and present our data.

Taxonomic assignment of representative sequences for both FL and V4 sequences were performed with the RDP (Ribosomal Database Project) classifier [47] based on SILVA database 138.1 version [48]. Those V4 sequences assigned to Archaea were discarded, since the FL sequences only captured Bacteria. The discarded sequences took up less than 5% of the total V4 sequences.

Representative sequences for both FL and V4 sequences were aligned by PyNAST [49] and phylogenetic trees were constructed with FastTree [50] (Fig. 1A). To eliminate the influence of differences in sequencing depth on downstream analyses, 12573 of FL sequences and 42910 of V4 sequences were randomly resampled. Rarefaction curves were constructed for the normalized data (Fig. S2). Taxonomic alpha diversity (Simpson index and observed richness) were calculated separately for each temperature group. In order to eliminate the influence of species richness on the microbial phylogenetic diversity, mean-nearest-taxon-distance (MNTD) and nearest-taxon-index (NTI) [51, 52] which independent of species richness were determined. They were calculated to reflect the phylogenetic distinctiveness of the tips of the tree using ‘mntd’ and ‘ses.mntd’ in ‘picante’ R package. MNTD was calculated as the mean of the branch lengths connecting each OTU to its closest relative within a sample and NTI as −1 times of the standardized effect size of MNTD accounting for the effects of species richness via 999 random resampling from a source pool based on a null model [53]. Smaller MNTD value represent closer phylogenetic relatedness among tips and higher NTI value indicated more phylogenetic clustering among tips [51, 54].

A phylogenetic tree for 1555 species with clear annotation at species-level was constructed and visualized using iTOL Pipeline (https://itol.embl.de/) [55]. Each species was represented by the most abundant OTU. Different colors for branches and the innermost ring indicate various Phyla, and the outermost ring represents the thermal tolerance breadth trait. The bargraphs showed the relative abundance of each OTU across temperature groups.

Blomberg’s K was calculated to represent the phylogenetic signal based on the environmental preferences of taxa as their potential traits using the “multiPhylosignal” function in the “picante” R package [56].

Averaged community-level niche breadth represented by Levins’ niche breadth index [57] was determined using the “spaa” package in R [58]. Larger niche breadth could indicate more available resources to microbial communities [59, 60].

To evaluate the dispersal capacity of community, we used a neutral community model (NCM) to predict the relationship between OTU detection frequency and their relative abundance across the wider metacommunity [61, 62]. NCM is an adaptation of the neutral theory adjusted to large microbial populations and usually used to quantify the importance of stochastic processes on community assembly. In this model, *Nm* is an estimate of dispersal between communities. The parameter *R*^2^ represents the overall fit to the neutral model [61, 62]. NCM was performed on Tutools platform (https://www.cloudtutu.com), a free online data analysis website.

Spearman correlation was employed to explore the correlation between environmental factors and α-diversity indices, and between environmental factors and the relative abundance of different phyla. The significant correlations were visualized with “ggcor” package in R. Analysis of Similarities (ANOSIM) [63], Multi Response Permutation Procedure (MRPP) [63] and Permutational Multivariate Analysis of Variance (PERMANOVA) [64, 65] were performed to determine any significant differences across temperature groups. PCoA based on unweighted UniFrac matrix was used to display microbial community structure changes across temperature groups. Mantel tests and CCA were used to estimate the effect of environmental factors on variation of microbial community structures. Random forest models used to evaluate the relative importance of environmental factors influencing microbial community structure (the first axis of PCoA) were performed with “randomForest” and “rfPermute” in R [66]. We proposed environmental extremes to consider both temperature and other environmental factors together by reducing the dimensionality of environmental factors by Principal Component Analysis (PCA). Except for the spearman correlation and random-forest analysis, all analyses were performed via Galaxy Pipeline (http://mem.rcees.ac.cn:8080) [36].

### The classification of “T-sensitive” and “T-resistant” species

We used “T-sensitive” and “T-resistant” to represent species those are adapted to narrow and wide thermal tolerance breadth in the geothermal ecosystem, respectively. The grouping criteria of defining “T-sensitive” and “T-resistant” species is obtained by comparing the observed distribution to the expected distribution derived from 100,000 permutations. We found that the enrichments of species at a specific temperature and those under five to eight temperatures (when *N*_observed species_ > *N*_expected species_), which means species occurring at a specific temperature and those occupying at least five temperatures were not randomly observed, but driven by deterministic factors. Based on this rationale, species found in a specific temperature were classified as T-sensitive species and those found in five to eight temperatures as T-resistant species. Then the T-resistant and T-sensitive as two evolutionary states were subjected to Binary-State Speciation and Extinction (BiSSE) model to calculate their speciation, extinction and transition rate [67].

### BISSE models and the unified phylogenetic tree used for BISSE models

The fossil record, which is the richest source of information on the evolutionary events behind extant communities, is mostly absent for Bacteria and Archaea [68] and researchers must use extant sequence data for evolutionary reconstructions [69]. BiSSE models make it feasible to study evolutionary features of extant microbial species. BiSSE model is a phylogenetic tree-based model and is able to calculate the two-state (binary) evolutionary character (i.e., speciation, extinction, and state-transition rates) of extant species [67]. The analysis was performed with the “diversitree” R package [70].

The BiSSE model would necessitate a more comprehensive phylogenetic tree of life. First, we conducted an analysis based on the All-Species Living Tree (LTP) from SILVA database. The All-Species Living Tree was downloaded from the SILVA database and the identified T-resistant and T-sensitive species were mapped to this tree using BLASTN (identity ≥ 98%, length ≥ 1000, *E*-value ≤ 1e − 5). Totally, 3241 representative sequences (26070 from the original) were preserved for T-sensitive species and 217 (524 from the original) for T-resistant species. We found that a lot of different OTUs matched the same species in the SILVA database. Therefore, a subtree containing 1063 mapped species was extracted from the LTP tree and linearized, allowing reconstruction of an ultrametric tree using the “ape” package in R. In addition, the special geochemical conditions cause hot springs to breed a large amount of “microbial dark matter” [71, 72]. The microbiota dwelling geothermal springs experience faster evolution rates [25, 73], which could result in new “microbial dark matter”. These “microbial dark matter” can’t be included in a relatively complete database (such as the SILVA database). Therefore, when the representative sequences in geothermal springs are compared with the SILVA database, there are only subset of sequences could find their close relatives in the All-Species Living Tree (LTP). Thus, it is not comprehensive to calculate the BISSE model with reference to the subtree of All-Species Living Tree (LTP) to determine the evolutionary characteristics of microorganisms in hot springs. Therefore, we used our own sequences to construct a unified phylogenetic tree by FastTree. The BiSSE model analysis was performed based on the LTP subtree and the self-built phylogenetic tree.

### BISSE analysis for species with various thermal tolerances

For each input linearized phylogenetic tree, diversitree was run twice: first to produce a heuristic starting point for the simulation by using starting.point.bisse function, and then to obtain the maximum likelihood estimate for the rate parameters by utilizing find.mle function. During the first round of estimation, T-resistant and T-sensitive species were constrained to have identical speciation rates and extinction rates. The second round was run with all rate parameters unconstrained, allowing T-resistant and T-sensitive species having different speciation and extinction rates. To assess the robustness of the final estimation, the Analysis of Variance (ANOVA) test was used to verify whether the constrained results (from the first round) were significantly different from the unconstrained results (from the second round).

### Applying Metabolic Theory of Ecology (MTE) to quantify diversification potential, environmental effect and their relative strength

Given the positive contribution of speciation and transition rate and the negative contribution of extinction rate to microbial diversity, we defined an index: diversification potential (DP) as follows:

$${{{{{\rm{DP}}}}}}\left( T \right) = \lambda + t - \mu$$

Where *λ*, *μ* and *t* represent speciation rate, extinction rate and transition rate, respectively, and obtained from BiSSE model.

Given the filtered out effect induced by increased environmental filtering, we use the extinction rate to represent the environmental-filtering potential (EP) following the equation of

$${{{{{\rm{EP}}}}}}\left( T \right) = \mu$$

Since DP and EP were related to temperature, metabolic theory of ecology (MTE) [6, 74] was employed to quantify the variations of DP, EP and their relative strength (RS_DP vs EP_) along the temperature axis. The equations are as follows:

$${{{{{\rm{DP}}}}}}\left( T \right) \propto e^{ - E_{{{{{\rm{DP}}}}}}/KT}$$

$${{{{{\rm{EP}}}}}}\left( T \right) \propto e^{ - E_{{{{{\rm{EP}}}}}}/KT}$$

$${{{{{\rm{RS}}}}}}_{{{{{{{{\mathrm{DP}}}}}}}}\,{{{{{\rm{vs}}}}}}\,{{{{{{{\mathrm{EP}}}}}}}}} = {{{{{\rm{DP}}}}}}\left( T \right)/{{{{{\rm{EP}}}}}}\left( T \right) \propto e^{E_{{{{{\rm{EP}}}}}} - E_{{{{{\rm{DP}}}}}}/KT}$$

where *K* is Boltzmann’s constant and *T* is absolute temperature in kelvin (K). The activation energy *E* equals the inverse number of slope in the linear regression.

## Results

### Consistency analysis of long-reads and short reads of 16S rRNA genes

High-throughput sequencing of 16S rRNA gene has been radically changing our view of microbial evolution and diversity. The combination of FL and fragmented 16S rRNA gene could mitigate the poor phylogenetic classification from fragmented sequence alone and low quality limitations from FL sequence alone. Variant sequencing depths were obtained for FL and V4 fragmented sequences. The number of high-quality reads ranged from 12,573 to 28,924 sequences per sample for FL sequences, whereas from 42,910 to 243,816 for V4 sequences. Rarefaction curves indicated that most of the diversity could be covered at the resampling depth of 42,910 for V4 sequences, while that for FL sequences did not reach saturation at the resampling depth of 12,573 (Fig. S2).

In order to compare the consistency between V4 and FL sequences, pairwise sequence alignments were conducted using BLASTN (Fig. 1B). At 97% identity level, 82.51% of the V4 sequences could be found in the FL sequences, and 83.96% of the FL sequences could be matched to the V4 sequences. Furthermore, we found that single sOTU (sub-operational-taxonomic-unit, equal to amplicon sequence variant (ASV)) of V4 sequences could match multiple OTUs of FL sequences (Fig. 1C), which indicated that FL sequences covered more comprehensive taxonomic profile with higher phylogenetic resolution than the V4 sequences. Therefore, most of the subsequent analyses mainly relied on FL sequences. However, since FL sequences were not as deep as shorter sequences (Fig. S2), some analyses were also compensated with V4 fragmented sequences.

### Environmental constraints on microbial composition

**Fig. 2:**
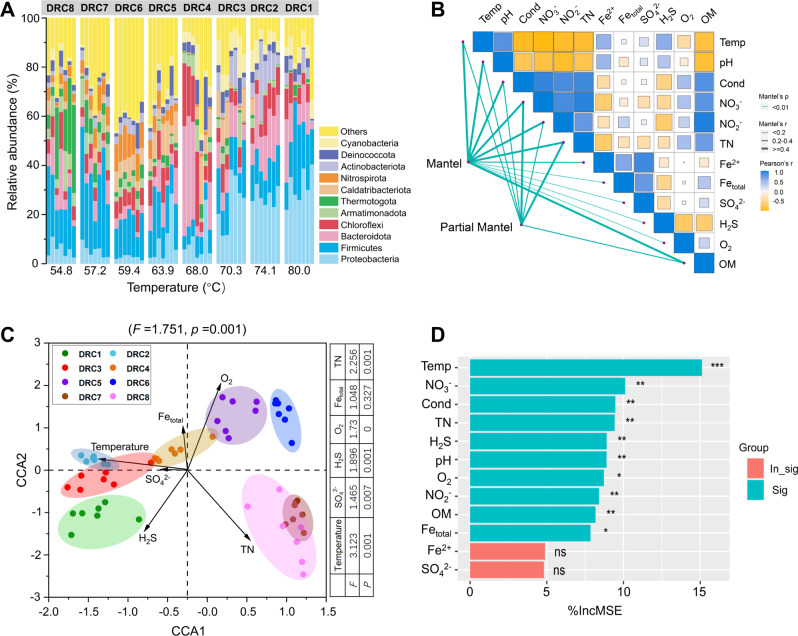
Microbial community variation and potential environmental constraints across sampling sites. **A** Microbial community composition at the phylum level, including 11 dominant bacterial phyla with average relative abundance greater than 3% in 56 samples, and those less than 3% were combined into “others”. The IDs of DRC8…DRC1 above the columns represent the eight sampling sites. **B**, **C** Significant correlations between environmental factors and community structure based on (Partial) Mantel test and canonical correspondence analysis (CCA), respectively. **D** The relative contribution of environmental factors to microbial community structure (the first axis of PCoA) based on random forest. %IncMSE means increase in mean squared error. The larger the value, the greater the importance of the environmental factor. *p* value, ns indicates non-significance; * for *p* < 0.05; ** for *p* < 0.01; *** for *p* < 0.001.

A total of 28,073 OTUs affiliated to 66 phyla were identified from FL sequences, with 11 dominant bacterial phyla (average relative abundance greater than 3% in 56 samples) accounting for 53.0–95.9% sequences in resampled samples. The relative abundance of dominant phyla fluctuated across temperature groups (Fig. 2A). Specifically, the relative abundance of *Proteobacteria*, *Bacteroidetes*, *Actinobacteria* and *Cyanobacteria* significantly increased (*p* < 0.05) with temperature, opposite to that of *Firmicutes*, *Armatimonadota*, *Thermotogota*, *Caldatribacteriota* and *Nitrospirota* (Fig. S3A). Microbial absolute abundance quantified by droplet digital PCR (ddPCR) decreased with temperature (Fig. S3B). Principal coordinate analysis (PCoA) showed that microbial community structures of different temperature groups were distinctly separated (Fig. S3C), as confirmed by the multiple dissimilarity tests (Table S2). The Mantel test showed that in addition to temperature, other variables such as pH, NO_3_^−^, NO_2_^−^, TN and OM co-varying with temperature also had significant (*p* = 0.001) associations with microbial community structures (Fig. 2B). Canonical correspondence analyses (CCA) further displayed a clear temperature-driven distribution pattern of microbial community structure (*F* = 1.751, *p* = 0.001) (Fig. 2C). In addition, Random Forest mean predictor importance of environmental variables indicated temperature was a more important predictor (higher MSE% value) driving microbial community structure pattern (Fig. 2D).

### High temperatures increased phylogenetic clustering

**Fig. 3:**
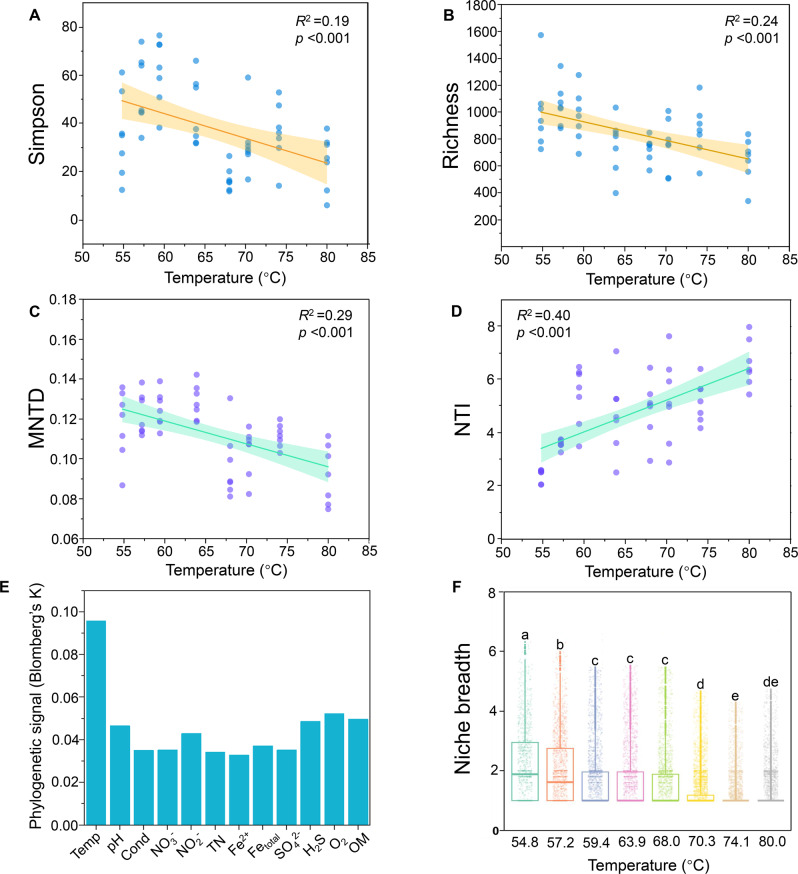
Microbial diversity patterns and community niche breadth across temperatures. Variation trends of **A** Shannon (*n* = 7), **B** Richness (n = 7), **C** Mean-nearest-taxon-distance (MNTD) (*n* = 7), **D** Nearest-taxon index (NTI) (*n* = 7) across temperatures. Pearson correlation coefficient was shown on the figure. **E** Phylogenetic signal estimated by Blomberg’s K and temperature shows the strongest phylogenetic signal. **F** Levins’ niche breadth across temperatures. The letters denote significant differences.

In order to decipher the microbial taxonomic and phylogenetic pattern across sampling sites, we examined Simpson’s diversity index, species richness, mean-nearest-taxon-distance (MNTD) and the nearest-taxon-index (NTI) along environmental extremes axis which represented the combined effect of environmental variables (Fig. S4). The geothermal springs are an analog to the ancient early earth. As we know earth experienced a process of cooling down and environmental temperature has been the most prominent factor driving the thermophile’s diversity expansion [21, 22]. Therefore, temperature was of particular interest in its effect on microbial diversity. We found the influence of temperature on microbial diversity (Fig. 3) was consistent with the influence of the combined effect of environmental variables (Fig. S4), indicating that temperature played a dominant role in driving microbial diversity pattern.

Specifically, Simpson’s diversity index decreased significantly with temperature (*R*^2^ = 0.19, *p* < 0.001) (Fig. 3A), from 35.0 ± 16.1 at 54.8 °C (DRC8) to 23.4 ± 10.5 at 80 °C (DRC1). A similar pattern was observed for species richness (*R*^2^ = 0.24, *p* < 0.001), which decreased from 996 ± 260 at 54.8 °C to 633 ± 150 at 80 °C (Fig. 3B). For phylogenetic patterns with temperature, MNTD decreased from 0.117 ± 0.016 at 54.8 °C to 0.091 ± 0.015 at 80 °C (*R*^2^ = 0.29, *p* < 0.001) (Fig. 3C), indicating that the phylogenetic relatedness became closer with temperature. Consistently, increasing NTI with temperature (*R*^2^ = 0.40, *p* < 0.001) was observed, from 2.412 ± 0.229 at 54.8 °C to 6.470 ± 0.642 at 80 °C (Fig. 3D), further suggesting higher temperature promoted stronger phylogenetic clustering at finer taxonomic level near the tips of the phylogenetic tree. These results were robust for V4 sequences with deeper sequencing depth (Fig. S5). Moreover, Blomberg’s *K* statistics revealed that phylogenetic signal for temperature is stronger than other environmental variables (Fig. 3E). Levins’ niche breadth became narrower with temperature (Fig. 3F). The Spearman correlation also confirmed the negative effects of temperature on Simpson’s diversity index, species richness, MNTD, biomass and Levins’ niche breadth (*p* < 0.05) and the positive effects on NTI (Fig. S6).

### Comparison of ecological properties between the T-sensitive and T-resistant species

**Fig. 4:**
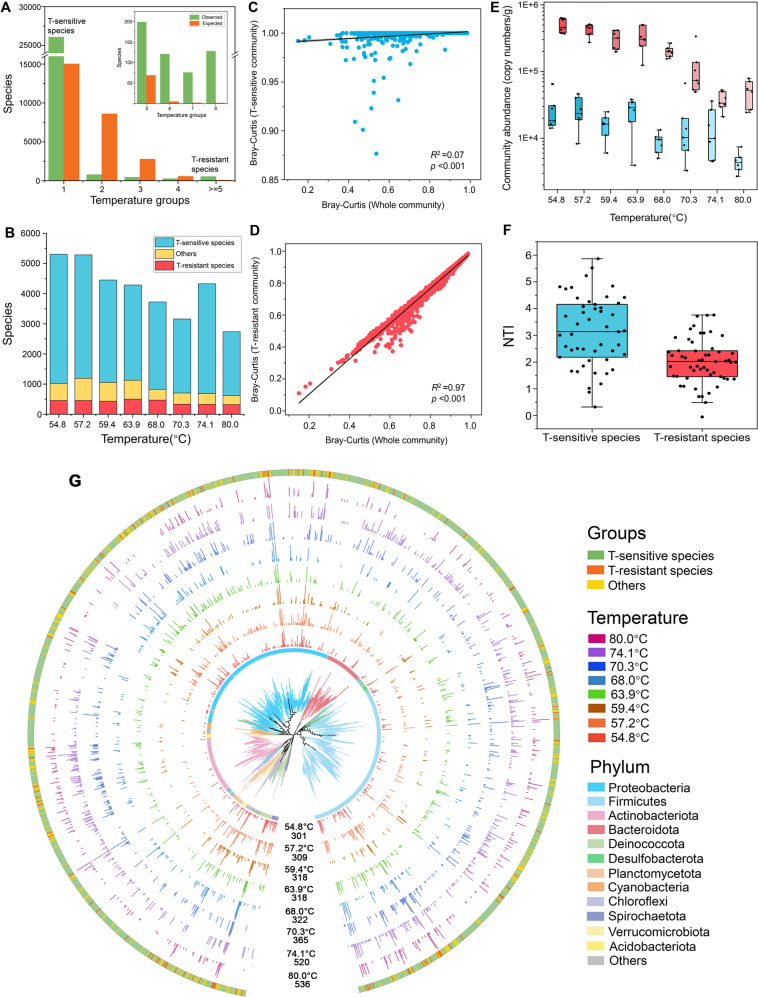
Ecological characteristics for thermal (T)-sensitive and T-resistant species. **A** Classification of T-sensitive and T-resistant species. The grouping criteria are obtained by comparing the observed distribution to the expected distribution derived from 100,000 permutations. We chose the grouping criteria when *N*_observed_ species > *N*_expected_ species, which means the species within the indicated temperature group are not randomly selected. In this study, the grouping criteria is 1 and ≥5, which is the rational for why we defined “T-sensitive” species as the species occurring at a specific temperature and “T-tolerant” species at least in five temperatures. The inset plot shows enrichments of species in five to eight temperatures. **B** The richness of T-sensitive and T-resistant species across temperatures. **C**, **D** The relationship of T-sensitive and T-resistant community structure with the whole community structure, respectively. **E** Variations in community abundance of T-sensitive and T-resistant species across temperatures quantified by ddPCR. **F** Variations in the within-community nearest-taxon index (NTI) between T-sensitive and T-resistant species. **G** The phylogenetic tree was constructed with 1555 species with clear taxonomic affiliations. Each species was represented by the most abundant OTU. Colors for both the branch and the innermost ring represent different Phyla, and colors for the outermost ring represent the thermal niche breadth (T-sensitive, T-resistant and others). The rings with inset bargraphs show the relative abundances of each OTU across temperatures. Temperature and species richness at each temperature were marked at the ends of the rings.

The T-sensitive and T-resistant species were identified according to narrow or wide thermal tolerance, respectively (Fig. 4A). A total of 26,070 phylotypes were classified as T-sensitive species (OTU-level species only found in a specific temperature) and 524 as T-resistant species (OTU-level species found in five to eight temperatures) (Fig. 4A). The T-sensitive species outnumbered the T-resistant species (2124–4286 vs. 318–455), both of which decreased from 54.8 °C to 80 °C with steeper decreasing trend for the T-sensitive species (Fig. 4B). Despite high species richness, T-sensitive species occupied much less community abundance (2.634 × 10^4^ vs. 4.620 × 10^5^, Fig. 4E) and sequences proportion (8.05% vs. 83.6%, Fig. S7A) than those T-resistant species. Additionally, the structures of T-sensitive sub-community showed greater dissimilarity between any paired temperature groups with little overlap to the whole community (Fig. 4C), but the T-resistant sub-community was significantly highly correlated to the whole community (Fig. 4D). However, compared to T-resistant species, T-sensitive species represented stronger phylogenetic clustering, with higher NTI (Fig. 4F). Consistently, T-sensitive species exhibited a narrower community-level Levins’ niche breadth (Fig. S7B) and a lower *Nm*-value (162 vs. 10429, Fig. S7C) of the neutral community model (NCM).

The phylogenetic tree was constructed with the representative sequences for species those having clear taxonomic affiliations (Fig. 4G). The relative abundance and the number of the species varied across temperature groups. From 54.8 °C to 80 °C, the richness of species was 301, 309, 318, 318, 322, 365, 520 and 536 respectively, showing a slightly increasing trend with temperature. The selected species were distributed in different phyla and most of the selected species belonged to T-sensitive species and a few to T-resistant species (1179 vs. 108).

### The evolutionary characteristics of the T-sensitive and T-resistant species

The variation trend of evolutionary characteristics (i.e., speciation, extinction, and state-transition rates) of T-sensitive and T-resistant species across temperature groups based on the LTP subtree (Table S3) were roughly as same as the unified phylogenetic tree based on our sequences (Table S4), so we only reported the results based on the self-built unified phylogenetic tree.

**Fig. 5:**
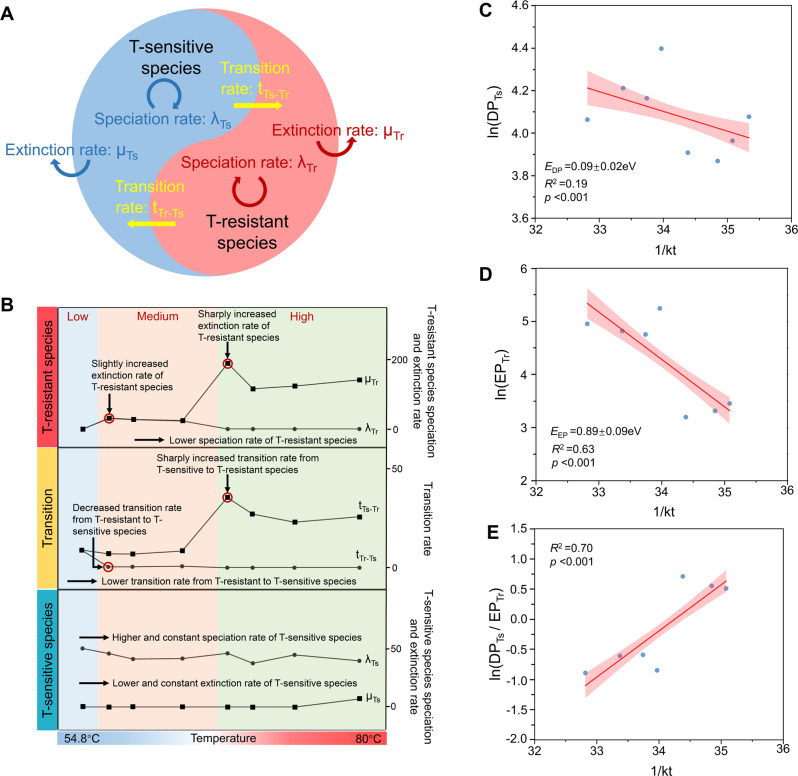
Evolutionary characteristics of T-sensitive and T-resistant species. **A** Binary-state speciation and extinction (BiSSE) model for the evolution of T-sensitive and T-resistant species. Each state has distinct speciation (*λ*), extinction (*μ*), and state-transition (*t*) rates. **B** The variation trend of the per species’ speciation rate (*λ*_Ts_ vs. *λ*_Tr_), extinction rate (*μ*_Ts_ vs. *μ*_Tr_) and transition rate (*t*_Ts-Tr_ vs. t_Tr-Ts_) for the T-sensitive and T-resistant species. **C**, **D**, **E** Effects of temperature, 1/kt, on the diversification potential of T-sensitive species (DP_Ts_, the slope *E*_DP_ = 0.09 eV), the ecological-filtering potential of T-resistant species (EP_Tr_, the slope *E*_EP_ = 0.89 eV) and the relative strength of DP_Ts_ to EP_Tr_ (RS_DP vs EP_), respectively. The linear line was fitted by using ordinary least-squares regression. The *X* axis was the reciprocal temperature (1/kT) and The *Y* axis was the ln-transformed DP_TS_, the ln -transformed EP_Tr_ and the ln-transformed DP_Ts_ to EP_Tr_, respectively.

The on average per species evolutionary rate parameters (i.e., speciation, extinction, and state-transition rates) of T-sensitive and T-resistant species across temperature groups were estimated using the BiSSE model with a maximum likelihood method (Fig. 5A). The results obtained by this model across temperature groups were reliable as proved by the significant difference between constrained and unconstrained results (ANOVA test, *p* < 0.001) (Table S4). The relatively low temperature of 54.8 °C favored speciation of T-sensitive lineages (speciation rate λ_Ts_ = 50.119), concomitant with a low but balanced reversible transition between T-sensitive and T-resistant lineages (*t*_Ts→Tr_ = 8.841 and *t*_Tr→Ts_ = 8.775) (Fig. 5B and Table S4). For the intermediate range of 57.2–63.9 °C, the most remarkable changes are that speciation (λ_Tr_) and extinction rates (*μ*_Tr_) for T-resistant species increased to 23.069–30.608 and 24.468–31.624, respectively (Fig. 5A, B and Table S4) and an advantageous transition from T-sensitive to T-resistant lineages (*t*_Ts→Tr_ = 6.829–8.333) than the reverse (*t*_Tr→Ts_= 0.406–0.738) (Fig. 5A, B and Table S4). For the high temperature range of 68–80 °C, the extinction rate of T-resistant species was further accelerated sharply (*μ*_Tr_ = 116.034–189.436), but the speciation rate (λ_Tr_) dropped back to 0 again (Fig. 5A, B and Table S4). High temperature favors more frequent transition to T-resistant lineages from T-sensitive lineages (*t*_Ts→Tr_ = 22.834–35.224) than the intermediate temperature. Notably, the extinction rate of T-sensitive lineages remained zero across temperature groups, but only peaked to 6.894 at an extremely higher temperature of 80 °C (Fig. 5A, B and Table S4).

Speciation (*λ*) and transition (*t*) rates act to increase microbial diversity, whereas extinction (*μ*) acts to decrease diversity. To evaluate the contributions of these processes to species richness, we propose a diversification potential (DP) index as DP = *λ* + *t* − *μ*. Given that increased temperature will filter out species, we use extinction rate to represent environmental-filtering potential (EP) as EP = *μ*. By applying DP and EP to T-sensitive and T-resistant species, we found that DP for T-sensitive species (DP_Ts_) and EP for T-resistant species (EP_Tr_) were both positive across temperatures, except for EP_Tr_ = 0 at the lowest temperature. More specifically, DP_Ts_ and EP_Tr_ both exponentially increased with temperature (*R*^2^ = 0.19, *p* < 0.001 (Fig. 5C) and *R*^2^ = 0.63, *p* < 0.001 (Fig. 5D)), with fitted activation energies of *E*_DP_ = 0.09 ± 0.02 eV (Fig. 5C) and *E*_EP_ = 0.89 ± 0.09 eV (Fig. 5D). Then, we assessed the relative strength of diversification potential versus environmental-filtering potential (RS_DP vs EP_) under the MTE framework. Specifically, RS_DP vs EP_ = DP_Ts_(*T*)/EP_Tr_(*T*) ∝ e^(*E*_EP_ – *E*_DP_)/KT (see derivation in “Materials and methods”). When *E*_DP_ > *E*_EP_, diversification overwhelms environmental filtering and overall diversity increases with temperature, else if *E*_DP_ < *E*_EP_, environmental filtering is advantageous over diversification and overall diversity decreases with temperature. Our results accorded well with the latter situation that *E*_DP_ < *E*_EP_ and RS_DP vs EP_ decreased exponentially with temperature (*R*^2^ = 0.70, *p* < 0.001) (Fig. 5E), indicating that environmental filtering is dominant over diversification at high temperatures.

## Discussion

Stronger environmental filtering and greater genomic diversification can exist in the same environmental context [6], but how these processes interact to influence microbial diversity across environmental gradients such as temperature was unclear. Here we chose appropriate sequencing and multivariate analysis methods to investigate the ecological and evolutionary characteristics of microbiota over a broad temperature range (54.8–80 °C). It’s a big challenge to reconstruct speciation and extinction processes of prokaryotes due to lack of fossil record [68]. By constructing relatively robust phylogenetic trees for the extant species, we could gain insights into evolutionary features of bacteria and archaea. In this study, we have some strategies to ensure the robustness of our data: (1) choosing the full-length 16S rRNA genes by PacBio RSII sequencing to obtain high microbial phylogenetic resolution [39]; (2) using NTI and MNTD which are controlling or eliminating the influence of species richness to calculate microbial phylogenetic pattern; (3) comparing the binary-state speciation and extinction model results from the LTP subtree and a unified phylogenetic tree based on our own sequences. Our results demonstrated that niche specialists and niche generalists cooperated to maintain microbial diversity via a dynamic equilibrium process, the underlying mechanism mainly including adaptive diversification of specialists, niche expansion of generalists and transition from specialists to generalists.

Species diversity is determined by both the physical (niche) and biological (biotic interaction) environments, both in ecological and evolutionary aspects. From ecological perspective, we previously found that temperature and interspecies interactions are deterministic factors affecting sediment community assembly [6]. In this study, several lines of evidence could support that temperature is the key environmental filter determining evolution: (1) the geothermal springs are analogous to the ancient early earth and environmental temperature has been a major determinant of evolutionary rates and the most prominent factor driving the thermophile’s diversity expansion during early earth cooling down [22]; (2) temperature was a more important predictor in driving the variation of microbial community structure revealed by higher random forest %MSE value (Fig. 2D); (3) there is strong clustering of phenotypes and the degree of clustering within communities is associated with temperature (Fig. 3C, D and Fig. S5); (4) temperature shows a higher phylogenetic signal than other environmental variables (Fig. 3E). Therefore, temperature could be the most important niche dimension in the studied geothermal ecosystems. By considering temperature niche axis, T-resistant (able to occur in at least five temperatures) and T-sensitive (only occupying a specific temperature) species essentially represent niche generalists and niche specialists in hot spring environments, respectively. We indeed found the discrepancy in ecological (Fig. 4) and evolutionary (Fig. 5) performances of these species with differential niche breadths. For instance, composition shift of T-sensitive species happened at a relatively fixed niche breadth (a temperature point) (Fig. 4C), whereas the composition turnover of T-resistant sub-community across temperatures was gradual and mirrored the whole community (Fig. 4D).

Niche specialization has long been argued to increase species speciation and adaptation rate [75], which could allow co-existence of more species via finer partitioning of limited niche space and resources [26]. Specifically, niche specialists (i.e. T-sensitive species) were strictly constrained by the local environment condition (limited space and resources) and experienced stronger dispersal limitation, as proved by the narrower Levins’ niche breadth (Fig. S7B) and negative value of *R*^2^ in the NCM (Table S5), as well as higher *Nm* across niche generalists (i.e. T-resistant) community (Fig. S7C). It is well known that niche expansion has come at cost of reduced capacity to adapt [76] and lower performance [77]; moreover, resource limitation enhanced speciation [78]. Therefore, the local endemism of niche specialists indicated maximum fitness at “home niche” and greater advantage in species diversification. Indeed, we observed higher speciation rate for T-sensitive species across temperatures (Fig. 5B and Table S4). Additionally, low abundance of T-sensitive species is beneficial for decreasing biotic interaction [79], and indirectly promoting high speciation and species richness. Given the more clustered phylogenetic relatedness of T-sensitive species than T-resistant species (Fig. 4F), T-sensitive lineages across temperatures may expand from phylogenetic closer species via sympatric speciation [80, 81] and thus each temperature accommodated more phylogenetically similar species, leading to increased competition among similar species under limited resources availability. However, low abundance reduced the physical contact of T-sensitive species with adjacent species and thus weakened competitive exclusion, allowing co-existence of more species with similar traits in a pretty narrow ecological niche (Fig. S7B), which eventually increased local diversity. Therefore, niche specialists were proposed to indirectly obtain higher speciation rates at the cost of less biomass and narrower niche breath, further promoting their relatively higher diversity.

Notably, despite of increasing extinction rate of T-resistant species, their species number was comparable across temperatures (Fig. 4B), mainly due to the concomitant increase in transition rate from T-sensitive to T-resistant species (Fig. 5 and Table S4). The continuous transition of T-sensitive species to T-resistant species ensures that the exclusion probability of T-resistant species is relatively constant at different temperatures. This transition of T-sensitive species to T-resistant species implied a “win-win” scenario between T-sensitive and T-resistant species: T-sensitive species (more constrained) need to achieve niche expansion via transition to T-resistant species (greater dispersal), while T-resistant obtained continuous replenishment since T-sensitive species generated relative stable species pool via high speciation and maintained a dynamic source-sink relationship with T-resistant species. Despite the differences in speciation and extinction rates between T-resistant and T-sensitive species, they are also evolutionarily related to each other. The findings of this very complex interaction and interdependence between the two have led to their co-evolution and co-adaptation, consistent with a key component of the Red Queen theory [82]. The balance of evolutionary dynamics between the niche specialists and niche generalists in hot springs could be the biological factor driving evolution.

**Fig. 6:**
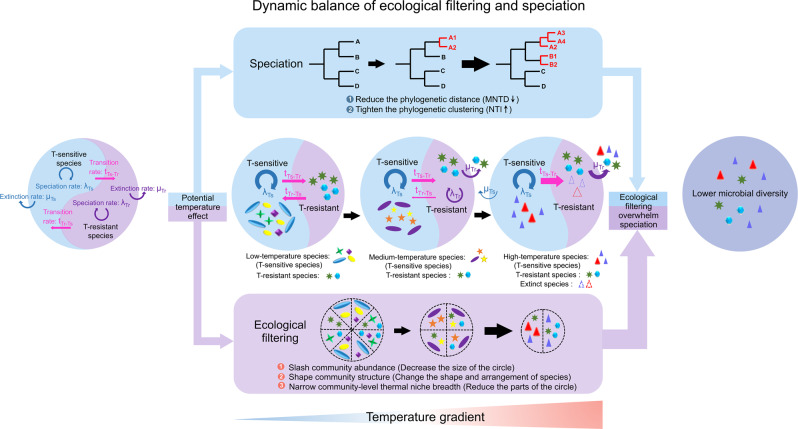
Conceptual diagram of the dynamic balance between microbial speciation and environmental filtering in a stressful environment such as high temperature. Higher temperature could enhance environmental filtering, resulting in less community abundance, reshaping community structure, and decreasing community-level thermal niche breadth. Simultaneously, higher temperature promotes speciation at finer tips of the tree, leading to reduced phylogenetic distance (i.e., MNTD) and strengthened phylogenetic clustering (i.e., NTI). When environmental filtering overwhelms speciation, we could observe a reduction in the microbial diversity pattern along the temperature gradient. The evolutionary characteristics underlying the consequential diversity pattern were determined by the dynamics of speciation, extinction, and transition rate for niche specialists (e.g., T-sensitive species only present at a narrow range of temperatures) and niche generalists (e.g., T-resistant species able to tolerate a wide range of temperatures) along an environmental extremes gradient (e.g. temperature).

We further elucidated relative contribution of environmental filtering and diversification to microbial diversity in response to a prominent niche axis (e.g., temperature in this study). The relative strength of diversification versus environmental filtering decreased exponentially with temperature (Fig. 5D), well in accordance with the overall reduction in diversity, indicating environmental filtering is advantageous over diversification when conditions became more stressful. We propose a conceptual framework to better describe the balancing of ecological and evolutionary processes in regulating diversity pattern along a prominent niche axis (e.g. temperature), (Fig. 6). Facing the intensive global change, microbes have been suffering more stressful conditions and this framework could be applied in other stressful environments and gain more deep understanding of how microbial diversity maintains in a phylogenetic aspect. Given the differences in niche breadth, dispersal ability and evolutionary characteristics, the transitions between niche specialists’ and niche generalists’ lifestyle help microbes adapt to environmental fluctuations.

## Supplementary information

High speciation rate of niche specialists in hot springs

## Data Availability

The sequencing data are deposited in the National Genomics Data Center (NGDC) database with accession numbers CRA007636 and CRA007773.
