# Supplementary material for: High speciation rate of niche specialists in hot springs
Source: ISME J. 2023 Jun 7;17(8):1303–14. doi: 10.1038/s41396-023-01447-4 (PMC10356836; doi:10.1038/s41396-023-01447-4)
Supplement: Supplementary file 1 — High speciation rate of niche specialists in hot springs [file 41396_2023_1447_MOESM1_ESM.docx]

**High speciation rate of niche specialists in hot springs**

Qing He^1,2^, Shang Wang^1*^, Kai Feng^1^, Sean T Michaletz^3^, Weiguo Hou^4^, Wenhui Zhang^4^, Fangru Li^4^, Yidi Zhang^4^, Danrui Wang^1,2^, Xi Peng^1,2^, Xingsheng Yang^1,2^, Ye Deng^1,2*^

^1^ CAS Key Laboratory for Environmental Biotechnology, Research Center for Eco-Environmental Sciences, Chinese Academy of Sciences (CAS), Beijing 100085, China

^2^ College of Resources and Environment, University of Chinese Academy of Sciences, Beijing 100190, China

^3^ Department of Botany and Biodiversity Research Centre, University of British Columbia, Vancouver, BC V6T 1Z4, Canada

^4^ State Key Laboratory of Biogeology and Environmental Geology, China University of Geosciences, Beijing 100083, China

Corresponding authors:

Shang Wang

CAS Key Laboratory for Environmental Biotechnology, Research Center for Eco-Environmental Science, Chinese Academy of Sciences, Beijing 100085, China

18 Shuangqing Road, Haidian District, Beijing 100085, China.

Telephone: 00 86 (010) 6284 0082

Fax: 00 86 (010) 6284 0082

E-mail: shangwang@rcees.ac.cn

Ye Deng

CAS Key Laboratory for Environmental Biotechnology, Research Center for Eco-Environmental Science, Chinese Academy of Sciences, Beijing 100085, China

18 Shuangqing Road, Haidian District, Beijing 100085, China.

Telephone: 00 86 (010) 6284 0082

Fax: 00 86 (010) 6284 0082

E-mail: yedeng@rcees.ac.cn


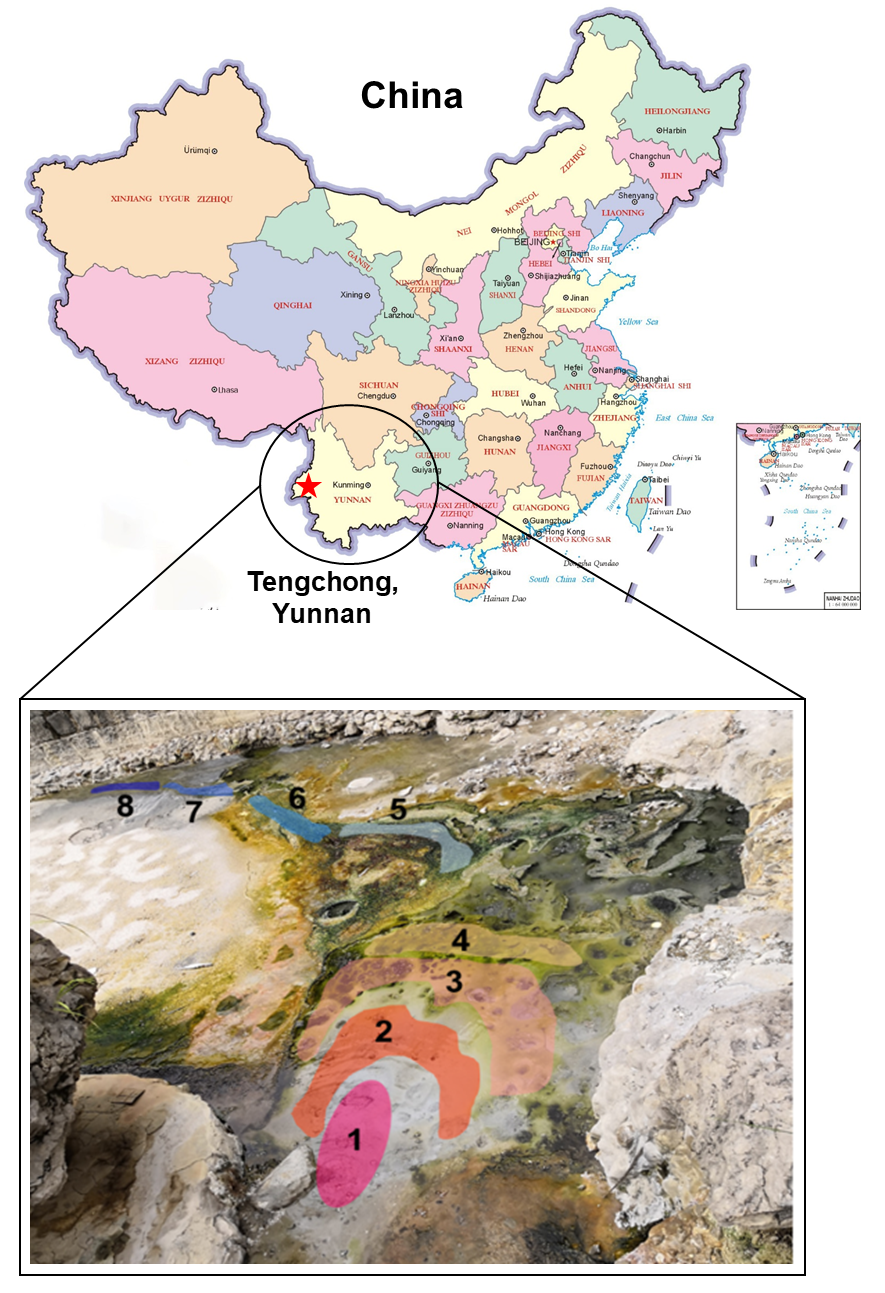
Fig.S1. Sampling map of DRC hot spring in Tengchong with a temperature gradient. Direchi (DRC) is an alkaline hot spring and has a certain temperature gradient (54.8℃-80℃) along the flow direction with the water spouting from the rock. According to the temperature gradient of DRC, we divided it into 8 different regions. DRC1 is the region with the highest temperature (80℃) because it is closest to the water jet of rock. With the flow of water, the temperature decreases gradually from DRC1 to DRC8, from 80℃ to 54.8℃.


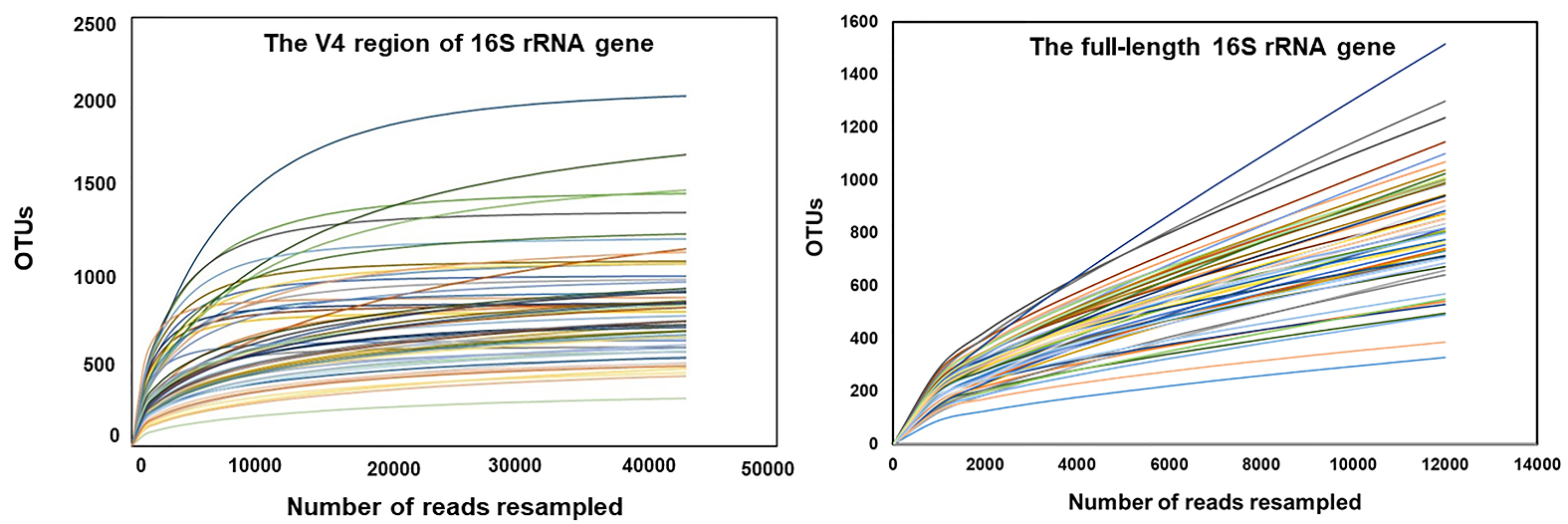
Fig.S2 The rarefaction curves constructed based on the number of resampled reads and the number of OTUs





Fig.S3 (A) Heat map indicating the correlations between environmental factors and the abundance of the 11 most dominant phyla. The circles shown in the figure are all significant correlations (*P*<0.05). Red and blue circles indicate positive and negative correlations, respectively. The size of the circles and the intensity of the color indicate the strength of the correlations and the values on the circles indicate the Spearman correlation coefficient. (B) Relationships between temperature and community abundance (n=7). (C) Microbial community structure by using principal coordinate analysis (PCoA) based on Unweighted-UniFrac distance.

Fig.S4 Relationships between environmental extremeness and (A) taxonomy diversity (n=7), (B) species richness (n=7), (C) mean-nearest-taxon-distance (MNTD) (n=7), (D) nearest-taxon index (NTI) (n=7). We proposed environmental extremes index by reducing the dimensionality of environmental factors by Principal Component Analysis (PCA). Uneven number of dots under per temperature groups was due to the overlap.


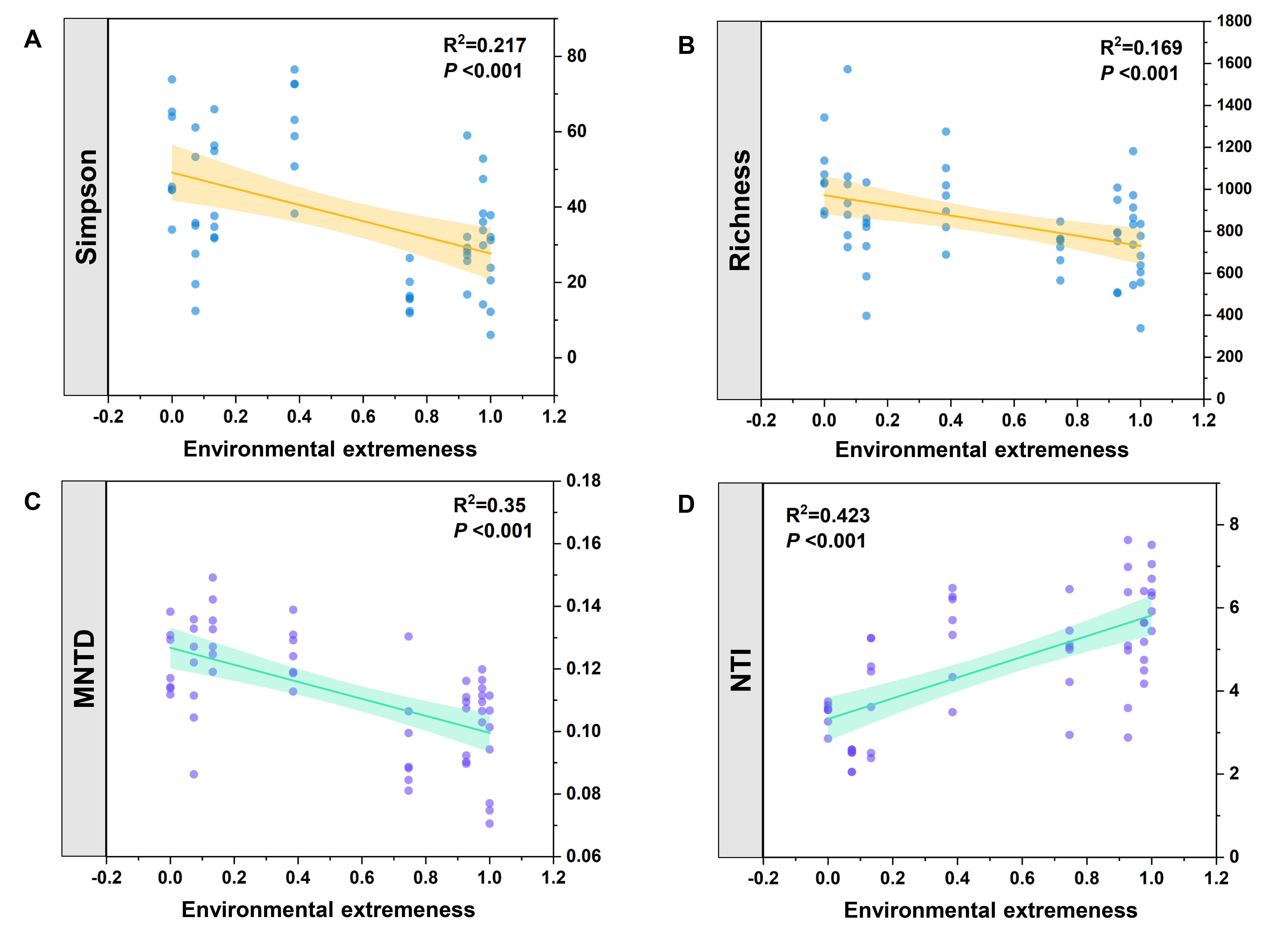


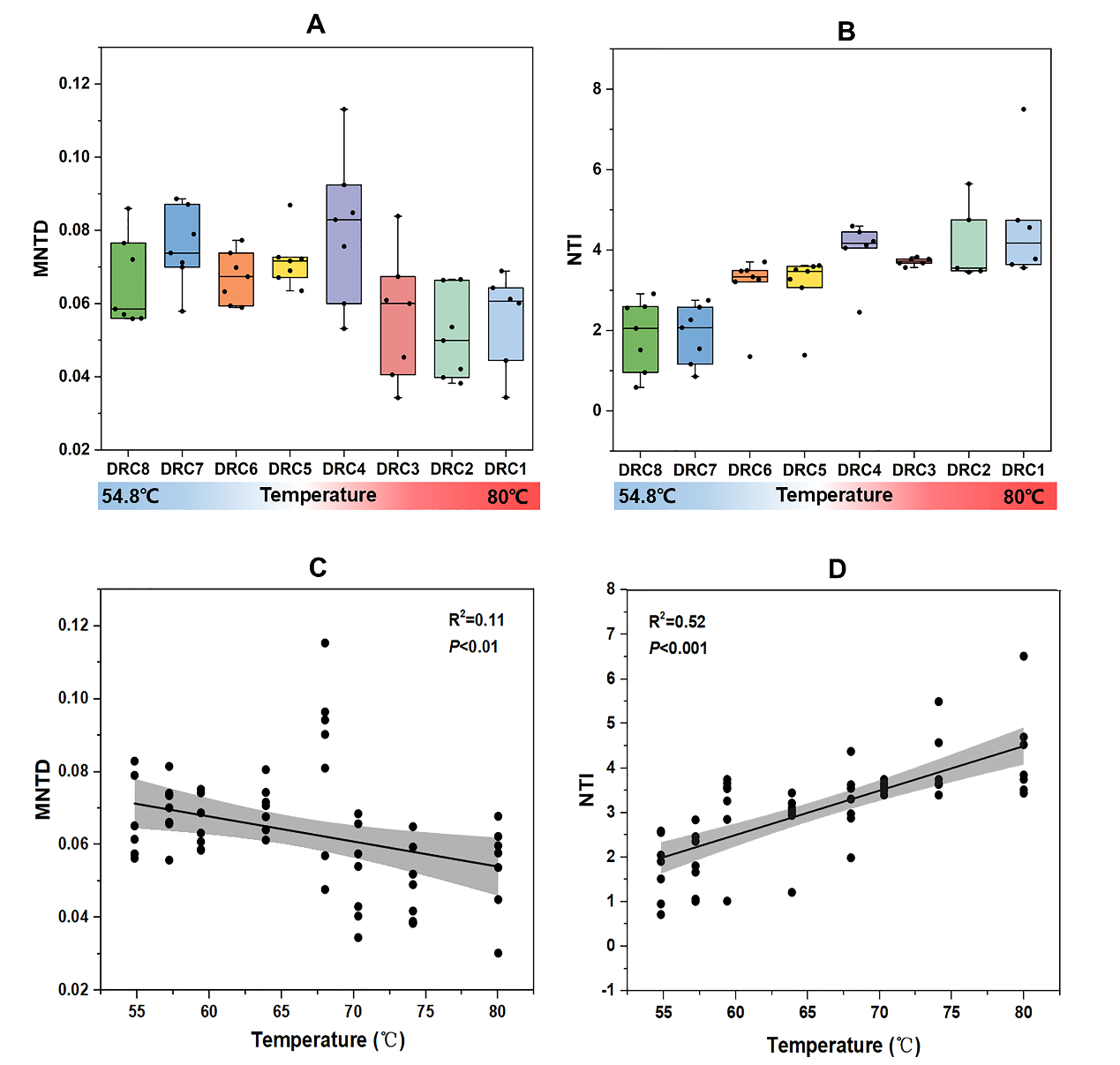


Fig.S5 (A) Variations of within-community mean-nearest-taxon-distance (MNTD) calculated based on NGS in the 8 temperature groups (n=7). (B) Variations of within-community nearest-taxon index (NTI) calculated based on NGS in the 8 temperature groups (n=7). (C) The relationship between mean-nearest-taxon-distance (MNTD) calculated based on NGS and temperature. (D) The relationship between nearest-taxon index (NTI) calculated based on NGS and temperature.


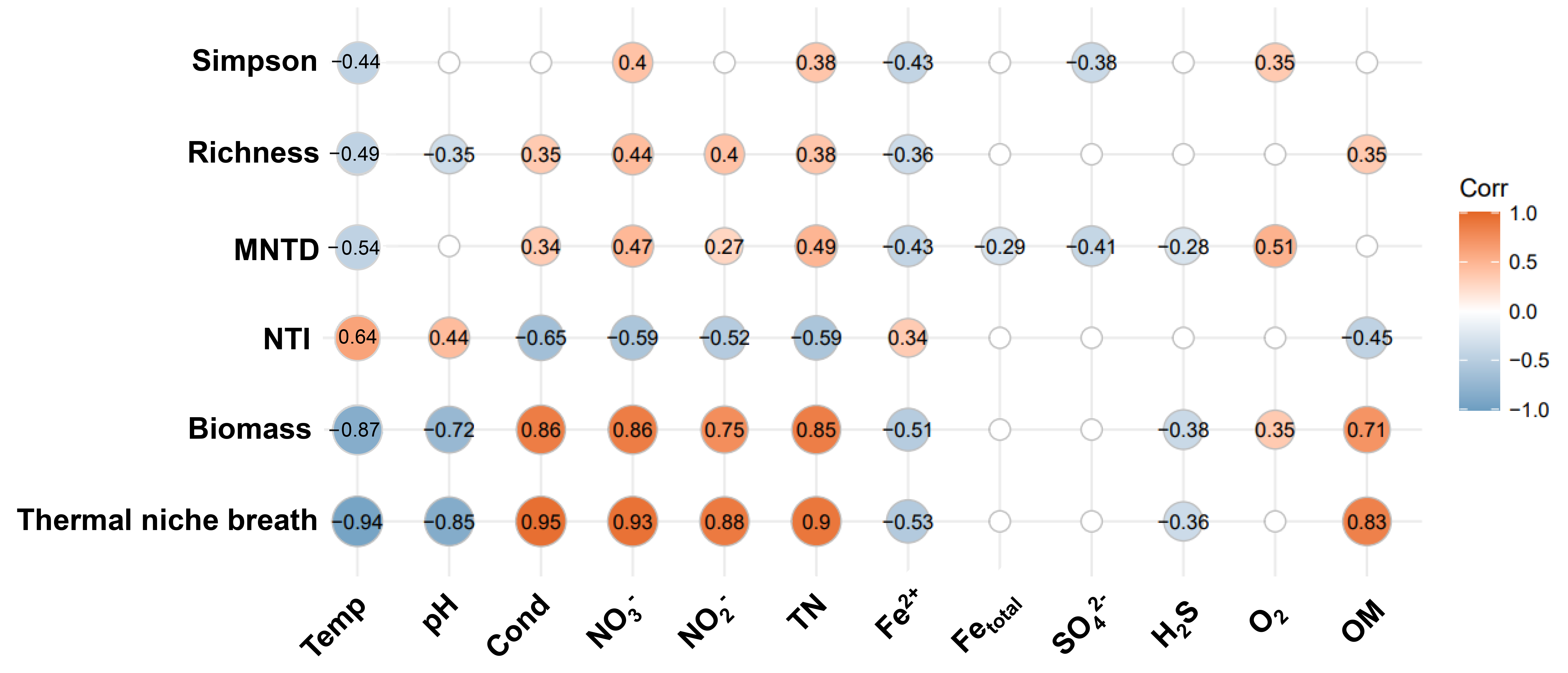


Fig.S6 Quantifying the environmental constraints on the simpson, richness, MNTD, NTI, biomass and thermal niche breath. The colored circles indicate significant correlations (P < 0.05), with red and blue for positive and negative correlations, respectively. The values on the circles indicate the Spearman correlation coefficient, with the size of the circles and the intensity of the colors proportional to the strength of the correlations.


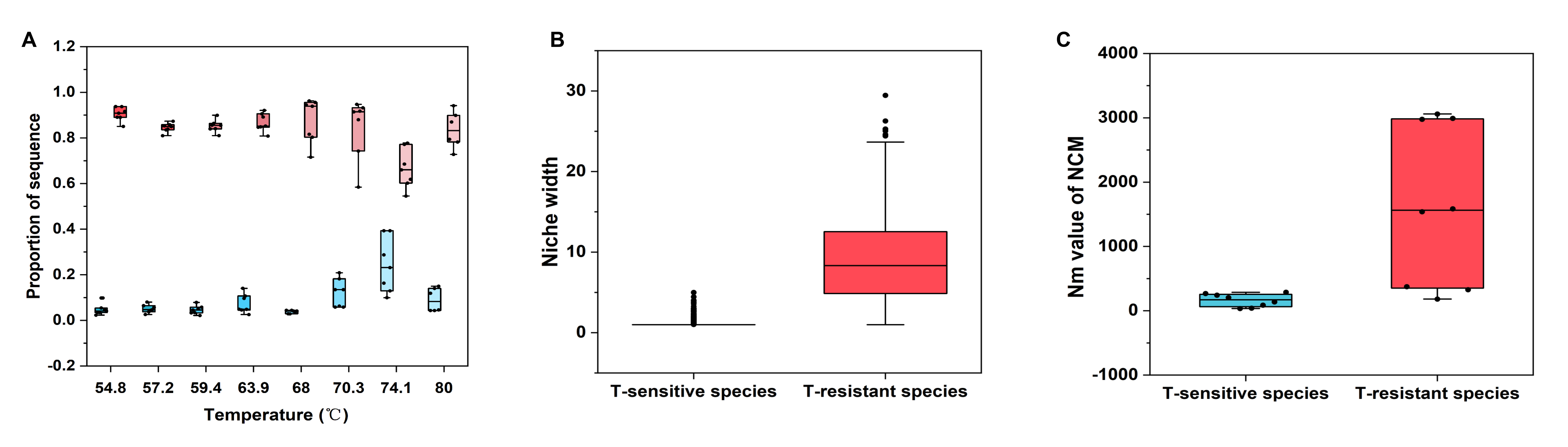


Fig.S7 (A) Variations in proportion of sequence of T-sensitive and T-resistant species following the temperature gradient. (B) Variations in thermal niche breath in all prokaryotic taxa in community between T-sensitive and T-resistant species. (C) Variations in Nm value of neutral community model (NCM) between T-sensitive and T-resistant species.

Table S1 Sample code, temperature, and environmental parameters of the temperature groups investigated in this study.

|  | **DRC1** | **DRC2** | **DRC3** | **DRC4** | **DRC5** | **DRC6** | **DRC7** | **DRC8** |
| --- | --- | --- | --- | --- | --- | --- | --- | --- |
| **Temperature**  **（℃）** | 80 | 74.1 | 70.3 | 68 | 63.9 | 59.4 | 57.2 | 54.8 |
| **pH** | 8.86 | 8.87 | 8.96 | 8.7 | 8.74 | 8.66 | 8.66 | 8.65 |
| **NO_3_^-^ （mg/L）** | 73 | 66 | 53 | 70 | 74 | 61 | 67 | 66 |
| **NO_2_^-^ （mg/L）** | 0.0605 | 0.0945 | 0.106 | 0.187 | 0.147 | 0.21 | 0.21 | 0.217 |
| **Fe^2+^**  **（mg/L）** | 0.277 | 0.257 | 0.533 | 0.527 | 0.213 | 0.193 | 0.187 | 0.133 |
| **SO_4_^2-^ （mg/L）** | 25 | 22 | 47 | 55.33 | 20.67 | 25.67 | 30.67 | 22.67 |
| **H_2_S**  **（mg/L）** | 0.71 | 0.45 | 0.257 | 0.26 | 0.34 | 0.223 | 0.347 | 0.363 |
| **O_2_**  **（mg/L）** | 6.1 | 7.6 | 9 | 8.1 | 10.1 | 8.8 | 8 | 8 |
| **Total iron （mg/L）** | 0.04 | 0.04 | 0.057 | 0.133 | 0.03 | 0.09 | 0.04 | 0.04 |
| **Conductivity （S/m）** | 0.99 | 1 | 1.13 | 1.16 | 1.21 | 1.19 | 1.26 | 1.32 |
| **Total N （mg/L）** | 229 | 236 | 269 | 364 | 684 | 552 | 754 | 715.13 |
| **Orgaic matter**  **（%）** | 0.37 | 0.4 | 0.5 | 0.78 | 0.61 | 0.78 | 0.85 | 0.76 |

Table S2 Dissimilarity tests of microbial communities for the 8 temperature groups. Groups with no significant (*P*>0.05) differences are shown in red.

|  | **MRPP** | | **ANOSIM** | | **PERMANOVA** | |
| --- | --- | --- | --- | --- | --- | --- |
|  | **r** | ***P*** | **r** | ***P*** | **r** | ***P*** |
| **DRC1 vs DRC2** | 0.6047 | 0.046 | 0.1817 | 0.032 | 1.833 | 0.048 |
| **DRC1 vs DRC3** | 0.6579 | 0.119 | 0.1457 | 0.084 | 1.4581 | 0.122 |
| **DRC1 vs DRC4** | 0.5585 | 0.002 | 0.7181 | 0.001 | 6.6001 | 0.001 |
| **DRC1 vs DRC5** | 0.5615 | 0.001 | 0.9718 | 0.002 | 9.6969 | 0.001 |
| **DRC1 vs DRC6** | 0.5706 | 0.002 | 0.9825 | 0.001 | 10.698 | 0.001 |
| **DRC1 vs DRC7** | 0.606 | 0.002 | 1 | 0.003 | 10.0425 | 0.001 |
| **DRC1 vs DRC8** | 0.5676 | 0.001 | 0.9902 | 0.003 | 10.7131 | 0.001 |
| **DRC2 vs DRC3** | 0.6401 | 0.077 | 0.1788 | 0.053 | 1.6715 | 0.063 |
| **DRC2 vs DRC4** | 0.5407 | 0.003 | 0.8474 | 0.001 | 9.4892 | 0.001 |
| **DRC2 vs DRC5** | 0.5436 | 0.001 | 0.9893 | 0.004 | 12.471 | 0.001 |
| **DRC2 vs DRC6** | 0.5528 | 0.001 | 0.9679 | 0.001 | 12.4046 | 0.003 |
| **DRC2 vs DRC7** | 0.5881 | 0.001 | 1 | 0.001 | 11.7718 | 0.001 |
| **DRC2 vs DRC8** | 0.5498 | 0.002 | 0.997 | 0.001 | 13.1361 | 0.002 |
| **DRC3 vs DRC4** | 0.5939 | 0.005 | 0.4586 | 0.007 | 4.5801 | 0.004 |
| **DRC3 vs DRC5** | 0.5968 | 0.001 | 0.7735 | 0.002 | 6.4305 | 0.001 |
| **DRC3 vs DRC6** | 0.606 | 0.001 | 0.8794 | 0.003 | 7.4785 | 0.001 |
| **DRC3 vs DRC7** | 0.6413 | 0.002 | 0.9834 | 0.003 | 7.3756 | 0.003 |
| **DRC3 vs DRC8** | 0.603 | 0.003 | 0.9339 | 0.001 | 8.4721 | 0.002 |
| **DRC4 vs DRC5** | 0.4974 | 0.003 | 0.6812 | 0.001 | 7.3147 | 0.004 |
| **DRC4 vs DRC6** | 0.5066 | 0.001 | 0.9115 | 0.003 | 12.092 | 0.001 |
| **DRC4 vs DRC7** | 0.5419 | 0.001 | 0.9543 | 0.001 | 10.5207 | 0.001 |
| **DRC4 vs DRC8** | 0.5036 | 0.002 | 0.8182 | 0.001 | 10.2458 | 0.001 |
| **DRC5 vs DRC6** | 0.5095 | 0.004 | 0.481 | 0.004 | 2.893 | 0.002 |
| **DRC5 vs DRC7** | 0.5449 | 0.002 | 0.8192 | 0.003 | 5.8782 | 0.001 |
| **DRC5 vs DRC8** | 0.5066 | 0.001 | 0.9407 | 0.001 | 9.5237 | 0.001 |
| **DRC6 vs DRC7** | 0.554 | 0.001 | 0.7016 | 0.002 | 4.2386 | 0.002 |
| **DRC6 vs DRC8** | 0.5157 | 0.001 | 0.9727 | 0.002 | 9.0948 | 0.002 |
| **DRC7 vs DRC8** | 0.5511 | 0.007 | 0.3197 | 0.006 | 3.1082 | 0.005 |

Table S3 Estimates of evolutionary characteristics for T-sensitive and T-resistant species across the temperature gradient. (Based on the subtree of the LTP tree)

| **Temperature Group** | | **T-sensitive species （T_s_）** | | | **T-resistant species （T_r_）** | | | **Conversion between T_s_ and T_r_** | | **ANOVA** |
| --- | --- | --- | --- | --- | --- | --- | --- | --- | --- | --- |
|  |  | **Number** | **Speciation rate** | **Extinction rate** | **Number** | **Speciation rate** | **Extinction rate** | **T_s_ to T_r_** | **T_r_ to T_s_** | **Chisq** |
| **80℃** | **High-**  **Temperature** | 131 | 7.124 | 0 | 35 | 0.137 | 38.26 | 13.391 | 0.886 | 16.179** |
| **74.1℃** |  | 265 | 9.934 | 0 | 37 | 0.082 | 35.471 | 12.478 | 0.738 | 36.719** |
| **70.3℃** |  | 180 | 9.034 | 0 | 34 | 0.024 | 34.896 | 16.461 | 0 | 11.838** |
| **68℃** |  | 65 | 6.595 | 0 | 36 | 0.069 | 26.326 | 14.876 | 0.553 | 6.59* |
| **63.9℃** | **Medium-**  **Temperature** | 119 | 7.898 | 0 | 38 | 5.637 | 6.307 | 9.514 | 1.127 | 7.03* |
| **59.4℃** |  | 68 | 5.864 | 0 | 33 | 3.027 | 4.88 | 3.505 | 1.449 | 4.3506* |
| **57.2℃** |  | 96 | 7.119 | 0 | 39 | 6.071 | 6.668 | 4.654 | 0.701 | 11.042** |
| **54.8℃** | **Low-**  **Temperature** | 87 | 7.058 | 0 | 39 | 0.117 | 0 | 4.318 | 3.33 | 29.117** |

**p*< 0.05; ***p*< 0.01; ****p*<0.001

Table S4 Estimates of evolutionary characteristics for T-sensitive and T-resistant species across the temperature gradient. (Based on own sequences to construct a unified phylogenetic tree by FastTreet)

| **Temperature Group** | | **T-sensitive species （T_s_）** | | | **T-resistant species （T_r_）** | | | **Conversion between T_s_ and T_r_** | | **ANOVA** |
| --- | --- | --- | --- | --- | --- | --- | --- | --- | --- | --- |
|  |  | **Number** | **Speciation rate** | **Extinction rate** | **Number** | **Speciation rate** | **Extinction rate** | **T_s_ to T_r_** | **T_r_ to T_s_** | **Chisq** |
| **80℃** | **High-**  **Temperature** | 2124 | 39.491 | 6.894 | 318 | 0 | 141.719 | 25.578 | 0 | 109.82*** |
| **74.1℃** |  | 3646 | 44.593 | 0 | 323 | 0 | 123.756 | 22.834 | 0 | 278.62*** |
| **70.3℃** |  | 2454 | 37.412 | 0 | 331 | 0 | 116.034 | 26.904 | 0 | 216.77*** |
| **68℃** |  | 2903 | 45.961 | 0 | 468 | 0 | 189.436 | 35.224 | 0.002 | 81.445*** |
| **63.9℃** | **Medium- Temperature** | 3163 | 41.457 | 0 | 497 | 23.069 | 24.468 | 8.333 | 0.738 | 116.2*** |
| **59.4℃** |  | 3397 | 41.043 | 0 | 430 | 26.893 | 27.551 | 6.829 | 0.455 | 92.268*** |
| **57.2℃** |  | 4097 | 45.697 | 0 | 455 | 30.608 | 31.624 | 6.969 | 0.406 | 190.77*** |
| **54.8℃** | **Low-Temperature** | 4286 | 50.119 | 0 | 455 | 0 | 0 | 8.841 | 8.775 | 291.31*** |

**p*< 0.05; ***p*< 0.01; ****p*<0.001

Table S5 The *R*^2^ and *Nm* value of the neutral community model (NCM) of T-sensitive and T-resistant species.

| **Temperature group** | **T-sensitive species** | | **T-resistant species** | |
| --- | --- | --- | --- | --- |
|  | ***R*^2^** | ***Nm*** | ***R*^2^** | ***Nm*** |
| **80℃** | -318.508 | 40 | -0.142 | 182 |
| **74.1℃** | -60.6 | 34 | 0.478 | 374 |
| **70.3℃** | -170.004 | 86 | 0.227 | 327 |
| **68℃** | -74.587 | 204 | 0.244 | 1585 |
| **63.9℃** | -86.074 | 138 | 0.473 | 1539 |
| **59.4℃** | -18.013 | 242 | 0.708 | 3059 |
| **57.2℃** | -8.486 | 288 | 0.672 | 2991 |
| **54.8℃** | -28.904 | 268 | 0.669 | 2977 |
